# The Rac3 GTPase in Neuronal Development, Neurodevelopmental Disorders, and Cancer

**DOI:** 10.3390/cells8091063

**Published:** 2019-09-11

**Authors:** Ivan de Curtis

**Affiliations:** Cell Adhesion Unit, Division of Neuroscience, San Raffaele Scientific Institute and Vita-Salute San Raffaele University, 20132 Milano, Italy; decurtis.ivan@hsr.it

**Keywords:** Rho family GTPases, Rac3, neuronal development, mutations, intellectual disability, cancer, invasion, metastasis

## Abstract

Rho family small guanosine triphosphatases (GTPases) are important regulators of the cytoskeleton, and are critical in many aspects of cellular and developmental biology, as well as in pathological processes such as intellectual disability and cancer. Of the three members of the family, Rac3 has a more restricted expression in normal tissues compared to the ubiquitous member of the family, Rac1. The Rac3 polypeptide is highly similar to Rac1, and orthologues of the gene for Rac3 have been found only in vertebrates, indicating the late appearance of this gene during evolution. Increasing evidence over the past few years indicates that Rac3 plays an important role in neuronal development and in tumor progression, with specificities that distinguish the functions of Rac3 from the established functions of Rac1 in these processes. Here, results highlighting the importance of Rac3 in distinct aspects of neuronal development and tumor cell biology are presented, in support of the non-redundant role of different members of the two Rac GTPases in physiological and pathological processes.

## 1. Introduction

**Figure 1:**
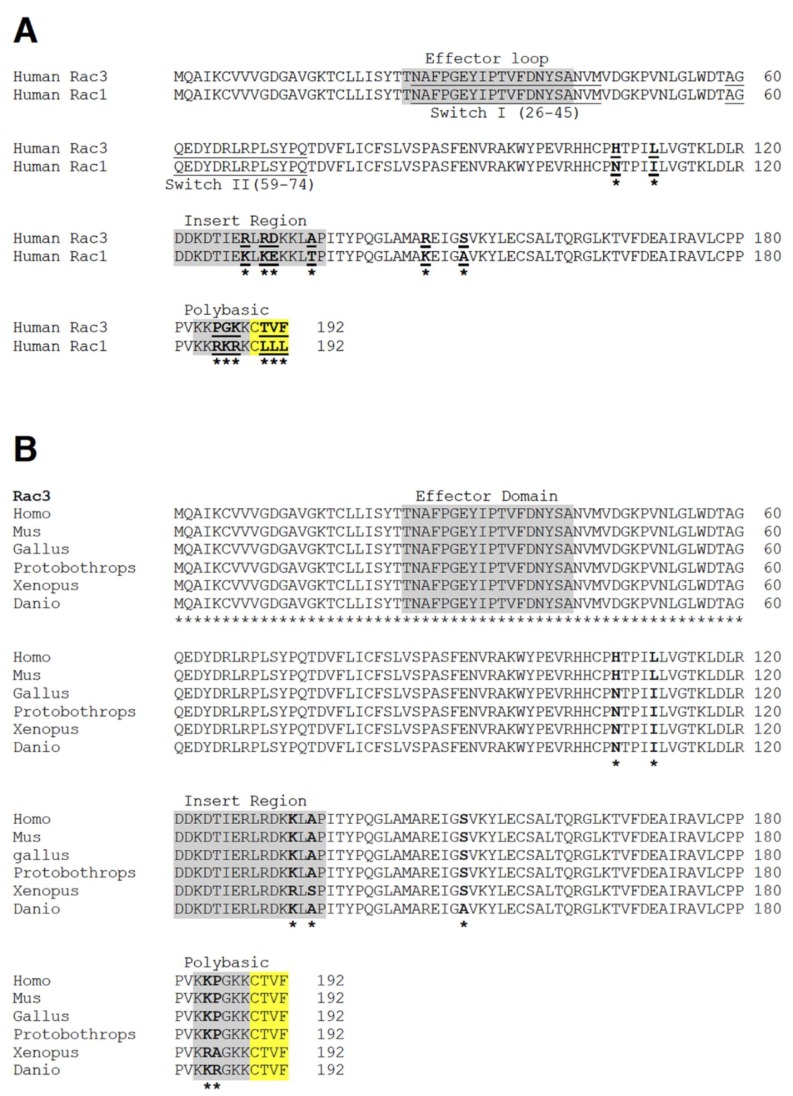
(**A**) Comparison of the primary sequence of human Rac1 and Rac3 proteins. (**B**) Comparison of the sequence of human and mouse Rac3 with Rac3 from a bird, a reptile, an amphibian, and a fish. Asterisks indicate residues differing among the aligned polypeptides. CAAX (C, Cysteine residue; A, aliphatic residue; X, any residue) boxes are in yellow.

In vertebrates the Rac family of guanosine triphosphatases (GTPases) includes three different genes that encode three highly similar proteins: the ubiquitously expressed Rac1 with its alternative spliced variant Rac1b [1,2]; Rac2 that is expressed only in hematopoietic cells [3]; and the Rac3 protein that is co-expressed with Rac1 in developing neurons and in other cell types, with a pattern of expression more restricted compared to Rac1 [4,5]. The three Rac proteins share high sequence similarity (88–92% identity) and diverge primarily in the last 10 carboxy-terminal residues (Figure 1A).

The Rac3 GTPase (not to be confused with the homonymous steroid/nuclear receptor-associated coactivator RAC3, also known as SRC-3, AIB1, ACTR, p/CIP, TRAM-1 [6]) is the last identified vertebrate Rac GTPase, first recognized in human cells [7] and in the chicken, where it was initially referred to as cRac1B [8] (Figure 1B). Rac3 has 92% amino acid identity with Rac1. The very carboxy-terminal end of the Rac proteins (residues 183–192) is the most divergent region between the Rac1 and Rac3 proteins (Figure 1A). This part includes a polybasic region and an adjacent CAAX box, where CAAX stands for the last 4 amino acid residues: a cysteine (C), followed by two aliphatic residues (AA), followed by any residue (X). The CAAX box is necessary for the post-translational modification of Rac proteins and is required together with the polybasic region for the subcellular localization and for the interaction of Rac GTPases with specific target proteins [9,10]. Interestingly, the CAAX motif of Rac3 is CTVF, which suggests that Rac3 may be a substrate for both geranylgeranyl transferase and farnesyl transferase [11].

The human gene for Rac3 maps to chromosome band 17q25.3 [7,12], near a region that is frequently deleted in breast cancer [13,14]. The gene for Rac3 has been found in the genome of several vertebrates, but not in lower phyla including lower chordates such as Ascidians (*Ciona intestinalis*) [15], Arthropoda or Nematodes. This review will present the growing experimental evidence that is pointing to important and unique functions of the Rac3 GTPase in neuronal development and cancer.

## 2. Rac3 in Neuronal Development

### 2.1. Expression of Rac3

Rac3 mRNA is expressed in different tissues, with higher levels in the nervous system including the human brain [7]. In chick neural tissues, the transcript for Rac3 is expressed in both the central and peripheral nervous system, and the protein level is developmentally regulated in the brain, reaching a peak at the time of intense neuritogenesis and synaptogenesis [8,16,17]. In situ hybridization on sections of the murine brain has confirmed the specific expression of Rac3 in several areas of the developing mammalian brain, where Rac3 is co-expressed with ubiquitous Rac1 [5]. In normal tissues, the Rac3 protein is detected prevalently in neural tissue [17]. Rac3 expression is evident in projection neurons in several regions of the developing brain and spinal cord, as well as in developing dorsal root ganglia [5].

### 2.2. Rac3 Specifically Influences the Maturation of Neurons in Culture

During brain development in mice, the expression of Rac3 appears to be developmentally regulated and more restricted compared to Rac1 [5], suggesting distinct roles of these highly similar GTPases. This is supported by the observation that the overexpression of Rac3, but not of Rac1, is potentiating the branching of neurites in cultured avian retinal neurons [16]. Interestingly, there are only 14 amino acid residues that differ between the two human Rac1 and Rac3 proteins (Figure 1A). Of these, six residues are found in the carboxy-terminal hypervariable region including the CAAX box that can be post-translationally prenylated in both the Rac1 and Rac3 proteins [18,19]. Interestingly, the use of chimeric constructs between the two GTPases has shown that the presence of the last eight carboxy-terminal residues of Rac3 is necessary and sufficient to induce increased neuritic branching. These results support the importance of the hypervariable region of Rac proteins for their specific effects, and suggest that this region of Rac3 may be involved in the interaction of the GTPase with specific protein partners, and/or in the localization at specific sites of the plasma membrane, to drive the extension of membrane protrusions.

Other differences have been detected in support of a specific role of Rac3 in spine formation in vitro. In contrast to hippocampal neurons depleted of Rac1, hippocampal neurons from Rac3 knockout mice develop in culture without showing obvious defects [20]. On the other hand, depletion of both Rac1 and Rac3 determine a much stronger phenotype compared to neurons lacking only Rac1; while Rac1 null neurons can still form dendritic spines, the formation of spines is strongly hampered in neurons lacking both Rac1 and Rac3 proteins [20]. Moreover, it has been recently observed that the re-expression of either Rac3 or Rac1 in double knockout mouse hippocampal neurons leads to distinct effects on the rescue of dendritic spines [21]. While Rac1 re-expression in double null neurons is significantly more efficient than Rac3 in restoring the formation of spines, the re-expression of Rac3 in double null neurons induces a more pronounced increase in the size of the spines compared to Rac1. This study highlights specific roles of Rac1 and Rac3, which may also be functionally relevant to synaptic plasticity. The enlargement of the spine head is a morphological event associated to synaptic potentiation [22]. Interestingly, in extracts from cortical neurons Rac3, but not Rac1, interacts specifically with the actin organizer β1-spectrin. The expression of the actin-binding domain (ABD) of β1-spectrin concentrates at dendritic spines, inducing the enlargement of the spine heads and the increase of the amplitude of miniature excitatory postsynaptic currents (mEPSCs) in hippocampal neurons, as observed after Rac3 expression [23]. These data suggest that β1-spectrin and Rac3 cooperate to modulate the morphological and functional dynamics of dendritic spines. Since the gene for Rac3 has appeared in vertebrates late during evolution [24], the findings suggest a possible specific role for this GTPase in the regulation of cognitive functions. This hypothesis is supported by recent findings that mutations in the Rac3 gene cause severe forms of intellectual disability in humans, as will be discussed in the next section.

### 2.3. Contribution of Rac3 to Mammalian Brain Function

As expected, the full deletion in mice of the gene for the ubiquitous Rac1 GTPase results in the early embryonic death of the knockout mice [25]. On the other hand, the conditional deletion of the gene for Rac1 in different populations of developing neurons and at different stages of development affects the development and behavior of the mutant mice to different degrees [26,27,28,29,30,31,32,33,34]. For example, the analysis of mice carrying the conditional deletion of Rac1 in the ventricular zone of the telencephalon with Foxg1-Cre has indicated that Rac1 may control axon guidance rather than neuritogenesis, since the mutant mice do not form the anterior commissure and show altered corpus callosal and hippocampal commissural axons [26]. On the other hand, the selective deletion of Rac1 in forebrain projection neurons by the CaMKIIα-Cre [29] impairs the learning-evoked increase in neurogenesis in the adult mouse hippocampus, revealing a Rac1-dependent signaling mechanism that stimulates the proliferation of new neurons generated during learning [34].

The full deletion of the gene for Rac3 does not affect the fertility and viability of Rac3 knockout mice [5]. Morphological analysis of these mice has not revealed evident defects in the organization of the brain. Still, the Rac3 knockout mice show differences in the performance of behavioral tasks that indicate cognitive alterations of adult Rac3 null mice. The expression of the Rac3 protein is highest in the brain of early postnatal mice. The use of a set of standard behavioral tests to assay the cognitive properties of the mutant mice has shown that although Rac3 knockout mice have normal spatial reference memory, they show reduced behavioral flexibility to novel situations, becoming hyperactive and hyper-reactive when exposed to new stimuli, as detected by applying a dark/light box, emergence and novel object tests [35]. These results have highlighted specific functions of Rac3 that cannot be compensated by the co-expressed homologous Rac1. Therefore, although no obvious anatomical abnormalities are detected, subtler defects may exist to explain the observed behavioral abnormalities.

Ubiquitous Rac1 and neuron-specific Rac3 GTPases are co-expressed in the developing mammalian brain. The importance of the contribution of Rac3 in mammalian brain function has become more evident in studies that have compared the effects of the single deletion of the gene for either Rac1 or Rac3, with the defects observed in double Rac1 and Rac3 knockout mice. Cre-mediated conditional deletion of Rac1 in neurons with the Synapsin-I Cre recombinase combined with the full knockout of neuron-specific Rac3 has been used to address the role of Rac GTPases in postmitotic neuronal development. The loss of both genes causes motor behavioral defects, epilepsy, and premature death of mice, while deletion of either GTPase produces less striking phenotypes [20]. Double knockout mice show the selective impairment of the hippocampal dorsal hilus and alteration of the hippocampal circuitry, with strongly reduced presynaptic input from dentate granule cells associated with a deficit of hilar mossy cells. Further investigation has indicated that Rac1 and Rac3 GTPases participate in the normal development of hilar mossy cells by regulating the migration of the mossy cell precursors toward the dorsal hilus [36]. These findings show that Rac1 and Rac3 play complementary roles during late stages of neuronal and brain development.

One interesting aspect is the role played by the two Rac GTPases in the development of cortical interneurons. The majority of cortical and hippocampal γ-aminobutyric acid (GABA)ergic interneurons are born in the medial and caudal ganglionic eminences located in the ventral telencephalon [37]. The GABAergic precursors then migrate tangentially toward their final destination in the cortex and hippocampus [38,39,40]. After reaching their destination, the interneurons migrate radially to distribute at their proper location within the layers of the cortex or hippocampus [41]. Two earlier studies addressing the role of Rac1 in the development of cortical interneurons have suggested that Rac1 is not directly implicated in the regulation of the development of postmitotic migrating interneurons [26,42]. In the first study, conditional gene-targeting has been used to delete Rac1 in populations of migrating neuronal precursors. Depletion of Rac1 in the subventricular zone of the ventral telencephalon, where medial and caudal ganglionic eminences give origin to most cortical and hippocampal GABAergic precursors, has been achieved by using Dlx5/6–Cre–internal ribosomal entry site–enhanced green fluorescent protein (Dlx5/6–CIE) mice [26]. Deletion of Rac1 in the subventricular zone does not produce visible defects in the tangential migration of the GABAergic interneurons, since Rac1/Dlx5/6–CIE mice have similar numbers and distributions of cortical interneurons positive for parvalbumin and calbindin. These findings have suggested that Rac1 is not necessary for the motile properties required for the tangential migration of the GABAergic precursors. In the second study, the early conditional ablation of Rac1 from Nkx2.1-positive GABAergic progenitors results in the impairment of their migration, with a consequent strong decrease of the GABAergic interneurons found in the postnatal cortex [42]. In the same study, the finding that later ablation of Rac1 from postmitotic GABAergic interneurons using Lhx6-Cre mice does not affect the development of the neocortex with normal migration of interneurons has suggested that Rac1 is required for the proliferation of the cortical GABAergic progenitors, but not for their later migration and postmitotic development. 

On the other hand, developing neurons often co-express both Rac1 and Rac3 during development. Further analysis of mice depleted of both GTPases has shown that Rac1 and Rac3 act together to play an important role for the proper migration and maturation of postmitotic cortical and hippocampal GABAergic interneurons, since their lack causes a dramatic loss of hippocampal and cortical parvalbumin-positive interneurons in the double knockout mice [43,44]. Interneurons lacking both Rac GTPases show cytoskeletal defects in vitro, with reduced leading processes and multipolar morphology [44], suggesting that the defect in migrating toward their final destination may be due to defects in cytoskeletal dynamics that need to be further investigated. Accordingly, Rac1/Rac3 depletion also importantly affects the maturation of interneurons that reach their final destination, with reduction of inhibitory GABAergic synapses in the hippocampal CA1 and in the cortical pyramidal cell layers [43]. These results and the observed alteration of the electrophysiological properties of the morphologically unaffected pyramidal neurons in the double knockout mice [43] show that Rac1 and Rac3 contribute synergistically to postmitotic development of specific populations of GABAergic cells.

Mice with double knockout of Rac1 (conditional) and Rac3 (Atoh1-Cre;Rac1flox/flox;Rac3-/-) in the cerebellar granule neurons (CGNs) show impaired tangential migration at E16.5, and increased apoptosis of CGNs at the deepest layer of the external granule layer [45]. The behavioral analysis of the double knockout animals reveals severe ataxic gait in these mice, while this behavioral phenotype is not detected in mice with conditional deletion of Rac1 (Atoh1-Cre;Rac1flox/flox) nor in Rac3 knockout mice. CGNs in double knockout animals differentiate normally until expression of p27kip1 and NeuN in the deep external granule layer at postnatal day 5. Explants from the knockout mice show impaired Map2-positive dendrites. These defects in tangential migration and differentiation of CGNs may be the cause of the observed decreased size of the cerebellum and of the agenesis of the medial internal granule layer, respectively. Rac deleted cells also exhibit lower levels of Mid1, a microtubule-associated E3 ubiquitin ligase, and impaired mTORC1 signaling, pointing to a novel Rac–Mid1–mTORC1 pathway that may regulate medial cerebellar development [45].

These studies demonstrate that Rac1 and Rac3 play a redundant role in the development and functional maturation of cortical GABAergic cells and CGNs. On the other hand, a finer analysis of the phenotypes of the single conditional Rac1 (by the Synapsin-I Cre) or full Rac3 knockout lines has revealed specific alterations that point to specific non-redundant functions of the two GTPases in neuronal development. Conditional deletion of Rac1 results in mice with greater hyperactivity and cognitive impairment compared to mice with the deletion of Rac3 [35,46], leading to more evident alteration of both the morphological and physiological properties of the inhibitory circuits in the Rac1 mutants. Although deletion of either Rac causes a similar reduction in hippocampal inhibitory terminals, only Rac1-deficient mice show a concomitant strong upregulation of cannabinoid receptor-1-positive terminals that may underlie the stronger functional defects in these mutants. The functional defects can be partially rescued by an antagonist of the cannabinoid receptors [46].

Electroporation-mediated transfer of knockdown vectors for Rac1 and Rac3 in newborn mice has recently been used to address the development of neonatal dentate granule cells in vivo [47]. Knockdown of either Rac3 or Rac1 results in frequently mispositioned neonatally born dentate granule cells, with partially different effects of the two GTPases on the differentiation of these cells, revealing the independent role of the two GTPases in regulating the proper localization and differentiation of dentate granule cells.

Future studies on the function of Rac1 and Rac3 in the development of the vertebrate nervous system is expected to highlight other examples of both redundant and non-redundant functions played by these important regulators of neuronal development.

## 3. Rac3 and Intellectual Disability

Different Rho family GTPases, together with their regulators and effectors, have been implicated in human intellectual disability [48]. Although more studies have recently appeared showing that Rac3 is important for neuronal and brain development, and that this GTPase is not always replaceable by Rac1 for its function, the interest in this GTPase has been underestimated so far. This is probably also due to the fact that the relevance of Rac3 for human health has been limited by the lack of evidence for its direct implication in human disease. The importance of Rac3 for human health and its link to intellectual disability has been recently highlighted by the identification of mutations of the gene for Rac3 that cause neurodevelopmental defects in human patients.

*De novo* missense variants in the gene for Rac1 have been recently associated to developmental disorders [49]. Moreover, screening for mutations in the three genes of the Rac family in cell lines and public databases has identified several missense mutations in the genes for Rac1 and Rac2, including variants at residues that activate Rac proteins found in a variety of human tumors and that promote transformation of human cells [50]. A recent study describes the use of a genome-wide sequencing screen that has led to the identification of three *de novo* missense variants in the human gene for Rac3 that cause phenotypes of severe intellectual disability and brain malformations in the five patients carrying the mutations [51]. The three identified mutations give rise to Rac3 proteins carrying the amino acid substitutions Pro29Leu, Gln61Leu, and Glu62Lys in the switch I and switch II regions of the Rac3 polypeptide, which are expected to be constitutively activated (Figure 1A). So far, only the Gln61Leu substitution has been shown to produce a constitutively activated Rac3 [19,52]. Moreover, previous experimental evidence supports a transforming effect of the three different Rac3 variants identified in this study, which have been all observed in databases of somatic variation in cancer [51].

Syndromic neurodevelopmental disorders and brain structural abnormalities originating from variants of the gene for Rac3 are supported by a second recent study, describing another *de novo* variant of the gene for Rac3 leading to a substitution of an evolutionarily conserved amino acid (Pro34Arg) within the switch I region of the GTPase [53]. This patient shows a severe developmental delay with intellectual disability, epilepsy, and brain dysplasia.

These novel findings indicate that mutations in Rac3 cause novel brain disorders, possibly induced by a mechanism of constitutive activation of the GTPase.

## 4. Rac3 in Cancer

Different members of the Rho family of GTPases have been involved in tumorigenesis and are important regulators of cellular functions like cell morphology, gene expression, cell proliferation, and apoptosis, which are relevant to cancer [54,55]. The role of Rac1 in tumor progression is well established; Rac1 is overexpressed in different types of tumors and is important for malignant transformation [56,57]. On the other hand, the implication of the closely related Rac3 GTPase in tumor progression is becoming evident by the experimental evidence that has been accumulating over the past years. Below are summarized the studies that have revealed the implication of Rac3 in tumor progression, and that have addressed some of the mechanisms by which this GTPase may act specifically in different types of tumor cells.

Rac3 is expressed in different types of human tumors including breast cancer, glioblastoma [58], prostate cancer, and lung adenocarcinoma. Analysis of the expression and mutation of the gene for Rac3 on a limited number of brain tumors has shown that the transcript for Rac3 is overexpressed in 19% of brain tumors (*n* = 26), while activating mutation occurs in 63% of brain tumors (*n* = 19) [59].

The sustained activation of Rac3 in transgenic mice expressing V12Rac3 in the mammary epithelium leads to an elevated phosphorylation of Pak1, impaired mammary gland physiology, and the formation of mammary gland lesions [60]. The first evidence of the critical and distinct implication of Rac3 on the invasive behavior of breast carcinoma cells and glioblastoma cells has been obtained by silencing the expression of either Rac3 or Rac1 by RNA interference [61]. Depletion of Rac1 inhibits lamellipodia formation, migration and invasion, with limited effects on cell proliferation and survival. Also, depletion of the Rac3 protein, which remarkably only makes up a very small fraction of total Rac in these cells, strongly inhibits cell invasion but does not affect lamellipodia formation and migration, nor proliferation. The differences observed by depleting either Rac3 or Rac1 may be due to the activation of distinct signaling pathways by the two GTPases. This hypothesis is supported by a study on the analysis of the consequences of the expression of different effector domain mutants on Rac1- or Rac3-induced transformation [62]. In this study, the authors have tested anchorage-independent growth and carried out pull-down assays for effector binding and serum response factor-induced transcription, and they describe differences between the recognition of specific effectors by the two GTPases; they find that the well-known Rac1 effectors mixed lineage kinases 2 and 3 bind very poorly to Rac3 compared to Rac1.

Rac3 overexpression is an indicator of poor prognosis in human breast tumors and correlates with invasive breast cancer [63]. Recurrence free survival was shorter and metastatic events higher among women with ERα—positive tumors that have increased expression of mRNA for Rac3, while the expression of the mRNA for Rac3 does not correlate with metastasis in ERα—negative tumor samples. Interestingly, in contrast to what is observed for Rac3, Rac1 mRNA expression does not correlate with an increase in metastasis or a decrease in recurrence free survival in ERα—positive tumors [63]. 

One of the mechanisms that may lead to the overexpression of Rac3 in breast cancer has been recently described. The gene KMT2D codes for a histone methyltransferase that acts as a transcriptional regulator. KMT2D is mutated in 6% of triple negative breast cancers and is strongly associated with poor survival [64]. Rac3 is one of the genes regulated by KMT2D, which becomes upregulated as a consequence of KMT2D mutations that lead to an abnormal protein that mimics a lack of expression of the KMT2D gene.

**Figure 2:**
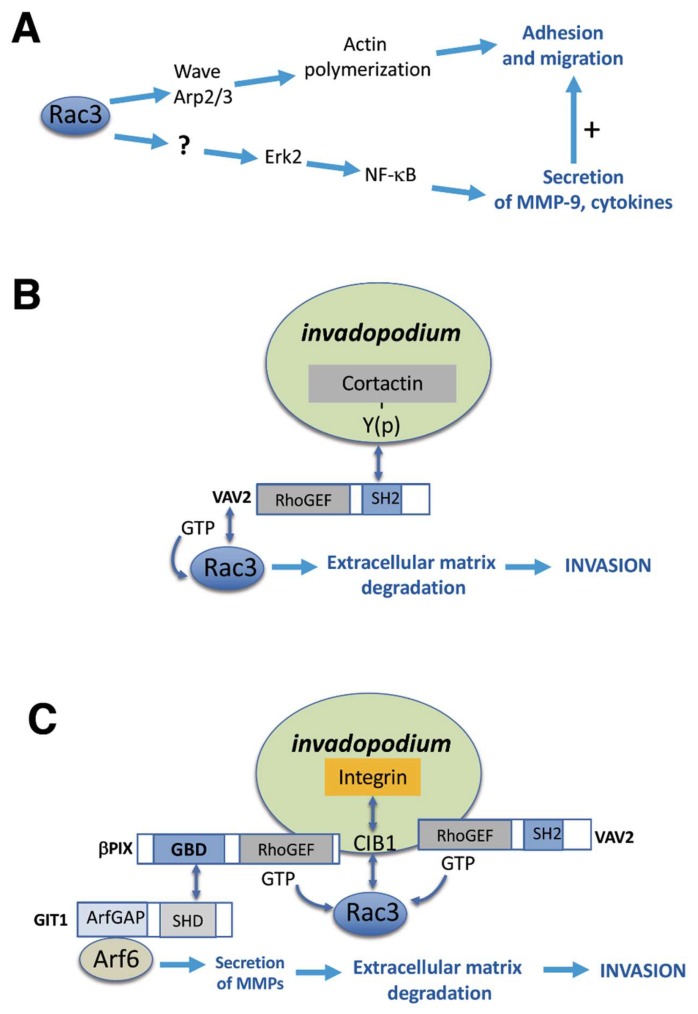
Rac3-mediated mechanisms in cancer. (**A**) Rac3-dependent aggressiveness depends on a pathway including Rac3, ERK2/mitogen-activated protein kinase 1 (MAPK1), and nuclear factor-κB (NF-κB) [65]. (**B**) Vav2 activates Rac3 at invadopodia to promote invasion [52]. (**C**) Model of cell signaling centering on Rac3. Rac3 contributes to balancing adhesion and extracellular matrix (ECM) degradation at invadopodia [79].

Rac3 depletion in invasive MDA-MB-231 breast cancer cells strongly reduces invasion and increases Tumor Necrosis Factor (TNF)-induced apoptosis, supporting a role for Rac3 in the aggressiveness of these tumor cells [65]. The analysis of the expression of signaling molecules downstream of Rac3 suggests that in MDA-MB-231 cells, the Rac3-dependent aggressiveness depends on a pathway including Rac3, the mitogen-activated protein kinase 1 (MAPK1/ ERK2), and nuclear factor-κB (NF-κB) that induces the secretion of the matrix metalloproteinase 9 (MMP-9) and of specific cytokines (Figure 2A), as well as resistance to TNF-induced apoptosis.

In non-invasive MCF-7 breast cancer cells, although Rac3 is expressed at similar levels to MDA-MB-231 cells, it does not support aggressiveness because the low expression of NF-κB subunits renders this pathway non-functional in these cells. These data indicate that Rac3 is an interesting therapeutic target to inhibit aggressive breast cancer.

Semiquantitative immunohistochemistry has been utilized to demonstrate that high-grade prostatic intra-epithelium neoplasia and prostate carcinomas express higher levels of Rac proteins when compared with benign prostate secretory epithelium [66]. All prostate carcinomas analyzed exhibit markedly higher Rac3 protein expression levels than the respective normal counterparts. Interestingly, on average there is a three-fold increase in the ratio of Rac3/Rac1 expression levels in all prostate carcinomas when compared with the respective normal counterparts [66]. One key process of the metastatic cascade in prostate cancer is extravasation (diapedesis) from the circulation into the bone [67]. Rac3 and Rac1 have opposite effects in PC-3 prostate tumor cell diapedesis; while depletion of Rac1 inhibits diapedesis, Rac3 knockdown promotes diapedesis, showing that Rac1 is required for PC-3 cell diapedesis across a bone marrow endothelial cell layer, while Rac3 inhibits tumor cell diapedesis [68].

Rac3 is a potential marker of invasion/metastasis and a therapeutic target also for lung adenocarcinoma. Indeed, Rac3 expression is significantly higher in lung adenocarcinoma compared with paired normal tissues, and the analysis of Rac3 expression in lung adenocarcinoma shows that positivity for Rac3 is associated with worse survival outcomes compared to Rac3 negative tumors [69]. In this study, Rac3 silencing inhibits lung adenocarcinoma cell migration and invasion in vitro and decreases the activity of the p38 MAPK pathway that is required for Rac3-induced migration and invasion, and for Rac3 regulated epithelial to mesenchymal transition. Silencing of Rac3 also inhibits proliferation and the formation of colonies by A549 lung cancer cells in vitro and induces a strong increase in tumor cell apoptosis [70].

Rac1, Rac2 and Rac3 are required for the growth, migration and invasion of glioblastoma cells [71]. Overexpression and downregulation of each endogenous Rac protein has been used to show similar effects of the three Rac proteins on glioblastoma cell lines. Rac1, Rac2 and Rac3 promote cell proliferation of glioblastoma cells that form tumor spheres, while these colonies are reduced in number and size by silencing either Rac GTPase. Silencing of either Rac1, Rac2 or Rac3 also inhibits glioblastoma cell migration and invasion in vitro. Moreover, the use of an in vivo zebrafish xenotransplantation model of human U373-MG glioblastoma cells has shown that silencing of either Rac GTPase promotes the survival of the fish embryos and abrogates angiogenesis induced by the xenotransplanted tumor cells [71].

Rac3 has also been implicated in promoting malignancies induced by the expression of the Bcr/Abl oncogene. The Bcr/Abl oncogene is a deregulated tyrosine kinase that causes chronic myelogenous leukemia and acute lymphoblastic leukemia in humans [72]. Rac3 is expressed in malignant lymphoblastic leukemia cells; activated Rac3, but not Rac1 or Rac2, can be detected in malignant precursor lymphoblasts from Bcr/Abl transgenic mice. Interestingly, crossing of Bcr/Abl transgenic mice with Rac3 knockout mice has shown that the lack of Rac3 is associated with longer survival of the transgenic animals, indicating a stimulatory role for Rac3 in leukemia in vivo [4]. Altogether, the results from the literature point to Rac3 as a possible marker and a new therapeutic target in aggressive tumors.

## 5. Mechanisms Underlying the Specificity of Rac3 Function

A number of studies have started to address the molecular mechanisms that may underlie the distinct effects played by Rac1 and Rac3 GTPases in different cellular environments. In neuroblastoma N1E-115 cells, Rac1 and Rac3 appear to play opposite roles, since depletion of Rac1 leads to decreased focal adhesions and cell rounding, while depletion of Rac3 induces stronger adhesions and growth of neurite-like protrusions. Accordingly, overexpression of Rac1 induces cell spreading, while overexpression of Rac3 induces cell rounding. Residues 185–187 in the variable polybasic region are responsible for the different phenotypes induced by the two GTPases, and for their different intracellular localization [73]. The analysis of the mechanisms underlying the specific effects of the two GTPases has shown that they both interact with the Arf6-GAP (GTPase activating protein) GIT1 [74], a regulator of cell matrix adhesion and spreading [75]. The Arf6-GAP activity of GIT1 is required for Rac3 signaling, and the data from this study suggest that Rac3 and Rac1 oppose each other’s function by differently modulating GIT1 signaling; Rac1 activates PAK1 and stimulates the GIT1–paxillin interaction, while Rac3 inactivates Arf6 by stimulating the Arf6-GAP activity of GIT1, thus preventing cell spreading [74].

Invasive tumor cells need to degrade the extracellular matrix (ECM) to be able to penetrate the surrounding tissues and start their dissemination for the formation of metastases. For this purpose, tumor cells often use adhesive structures called invadopodia, which can secrete different types of proteases to degrade the ECM [76]. Signaling activated by epidermal growth factor (EGF) triggers the cortactin-mediated maturation of invadopodia that are required for invasion by different aggressive tumor cells, including breast cancer cells [77]. Vav2 is a guanine nucleotide exchange factor that activates Rho family GTPases [78] and binds via its SH2 domain to phosphorylated cortactin to support ECM degradation by invasive MDA-MB-231 breast cancer cells [52]. The positive effects of Vav2 on invadopodia maturation, ECM degradation, and invasion depend on the activation of Rac3 at invadopodia, as shown by the inhibition of ECM degradation by Rac3 knockdown and the rescue of the negative effects of Vav2 knockdown on invadopodia function by a constitutively active Rac3 [52] (Figure 2B).

Rac3 is an important driver of invasion and formation of metastasis *in vivo*, as shown by the strong reduction of lung metastases in a mouse model injected with Rac3 knockout breast cancer cells compared to control cells expressing normal Rac3 levels [79]. The same study has shown that in breast cancer cells, the activity of Rac3 is critical for integrating the integrin-mediated invadopodia adhesion to the ECM, with the delivery of metalloproteases at invadopodia to degrade the ECM [79]. The activity of Rac3, detected by an optimized Fluorescence Resonance Energy Transfer (FRET) biosensor, is recruited at invadopodia by the Rac3-binding partner CIB1 [80], and is spatially and temporally regulated by the guanine nucleotide exchange factors Vav2 and βPIX (p21-activated protein kinase exchange factor), which affect the localization of the Arf6-GAP GIT1 [79]. GIT1 and the Arf6 GTPase regulate the vesicular trafficking required for ECM degradation. By regulating this signaling network, Rac3 participates in balancing adhesion with the proteolytic activity at invadopodia to optimize tumor cell invasion (Figure 2C).

Mammalian Rac1 and Rac3 share over 90% identity in their amino acid sequence [7], and the sequence of their effector loops (to which most Rac effectors bind) is identical (Figure 1A). Therefore, it is not clear how specificity is generated. The substantially different region between Rac1 and Rac3 is their C-terminal end, which includes elements that are important for the subcellular localization of the GTPases. Interestingly, the ubiquitous protein calcium- and integrin-binding protein 1 (CIB1) that was first identified as a cytoplasmic binding partner of platelet-specific integrin α_IIb_β_3_ [81], interacts specifically with an extended carboxy-terminal part of Rac3 (residues 145–192), but not with the corresponding region of Rac1 [80]. In mammalian cells, CIB1 interacts with activated Rac3 via its C-terminal end, but not with activated Rac1 or Rac2. The functional specificity of this interaction is supported by the finding that adhesion of cells to fibrinogen induces a specific increase of Rac3, but not Rac1 or Rac2, in the Triton-insoluble cytoskeletal fraction, and that cell adhesion via the α_IIb_β_3_ integrin leads to the increase of endogenous active GTP-Rac3 but not GTP-Rac1, implicating Rac3 and CIB in integrin-associated cytoskeletal reorganization during adhesion.

The Rac3 GTPase has been identified as a specific negative regulator of autophagy. Knockdown of Rac3, but not of Rac1 or Rac2, induces autophagy in different tumor cell lines, including prostate cancer PC3 and breast cancer MDA-MB-231 cells that express low levels of Rac3 [82]. Moreover, the ectopic expression of Rac3 can rescue cells from autophagy and cell death induced by inhibiting the carboxymethyl transferase enzyme that catalyzes the final step of prenylation of the CAAX box of several Rho family proteins, including Rac3.

The levels of Rac3 protein in the cell can be regulated by ubiquitination and proteasome degradation. F-box proteins are subunits of E3 ubiquitin ligases that recognize specific substrates for ubiquitination and degradation in the proteasome [83,84]. The F-box protein FBXL19 regulates Rac3 ubiquitination and stability. Rac3 supports Transforming Growth Factor (TGF)β1–induced E-cadherin down-regulation in esophageal cancer cells that is associated with poor prognosis in esophageal cancer [85]. The overexpression of FBXL19 decreases the expression of Rac3 by inducing its degradation; by antagonizing Rac3, FBXL19 affects TGFβ1-induced E-cadherin down-regulation. Thus, Rac3 represents a potential target to suppress TGFβ1–induced esophageal tumorigenesis.

## 6. Conclusions

The data presented in this review highlight the emerging roles of the Rac3 GTPase in normal development, and the recent implication in neurodevelopmental human disease, as well as in different types of cancers. These results highlight Rac3 as a potential biomarker of tumorigenesis and metastasis for different types of aggressive tumors, and as a promising therapeutic target for cancer and possibly neurodevelopmental disorders. The investigations over the past few years have highlighted several differences in the cellular processes and in the molecular mechanisms underlying the specific effects of Rac3 versus the ubiquitous Rac1. What is becoming evident is that in cells and tissues co-expressing Rac1 and Rac3 GTPases, the function of these proteins cannot be simply explained by functional redundancy, since an increasing number of examples are demonstrating specific effects of Rac3 that cannot be accomplished by Rac1. This is quite intriguing, especially considering the fact that Rac3 is often co-expressed at much lower levels compared to the ubiquitous Rac1. Some authors have proposed that hyperactivation of Rac3 may explain the strength of its effect even at lower levels of expression, although a conclusive demonstration that Rac3 is hyperactive in cells is still missing. On the other hand, the very limited but significant differences in amino acid sequence concentrated at hot spots along the Rac1 and Rac3 polypeptides suggest that these differences may drive the interaction with specific effectors/regulators for each GTPase, and/or guide their subcellular localization at specific locations within the cell. In this direction, it has been shown that the differences in the polybasic region at the carboxy-terminus of the two GTPases may explain the specific ability of Rac1, but not Rac3 and Rac2, to interact with regions of the membrane enriched in the phospholipid phosphatidylserine, which is recognized by the highly charged polybasic motif of Rac1, and is required for the morphological changes that support Rac1-promoted cell migration [86]. More work on the specificity of Rac3 subcellular localization and on the molecular mechanisms distinctly affected by either GTPase may also help us to understand the need for the late appearance during evolution of a third member of the vertebrate Rac family.

## References

[1] Moll J., Sansig G., Fattori E., van der Putten H. (1991). The murine rac1 gene: cDNA cloning, tissue distribution and regulated expression of rac1 mRNA by disassembly of actin microfilaments. Oncogene.

[2] Jordan P., Brazåo R., Boavida M.G., Gespach C., Chastre E. (1999). Cloning of a novel human Rac1b splice variant with increased expression in colorectal tumors. Oncogene.

[3] Didsbury J., Weber R.F., Bokoch G.M., Evans T., Snyderman R. (1989). Rac, a novel ras-related family of proteins that are botulinum toxin substrates. J. Biol. Chem..

[4] Cho Y.J., Zhang B., Kaartinen V., Haataja L., de Curtis I., Groffen J., Heisterkamp N. (2005). Generation of Rac3 null mutant mice: Role of Rac3 in Bcr/Abl-caused lymphoblastic leukemia. Mol. Cell Biol..

[5] Corbetta S., Gualdoni S., Albertinazzi C., Paris S., Croci L., Consalez G.G., de Curtis I. (2005). Generation and characterization of Rac3 knockout mice. Mol. Cell Biol..

[6] Li H., Gomes P.J., Chen J.D. (1997). RAC3, a steroid/nuclear receptor-associated coactivator that is related to SRC-1 and TIF2. Proc. Natl. Acad. Sci. USA.

[7] Haataja L., Groffen J., Heisterkamp N. (1997). Characterization of RAC3, a novel member of the Rho family. J. Biol. Chem..

[8] Malosio M.L., Gilardelli D., Paris S., Albertinazzi C., de Curtis I. (1997). Differential expression of distinct members of the Rho family of GTP-binding proteins during neuronal development: Identification of Rac1B, a new neural-specific member of the family. J. Neurosci..

[9] Kinsella B.T., Erdman R.A., Maltese W.A. (1991). Carboxyl-terminal isoprenylation of ras-related GTP-binding proteins encoded by rac1, rac2, and ralA. J. Biol. Chem..

[10] Ando S., Kaibuchi K., Sasaki T., Hiraoka K., Nishiyama T., Mizuno T., Asada M., Nunoi H., Matsuda I., Matsuura Y. (1992). Post-translational processing of Rac p21s is important both for their interaction with the GDP/GTP exchange proteins and for their activation of NADPH oxidase. J. Biol. Chem..

[11] Moores S.L., Schaber M.D., Mosser S.D., Rands E., O’Hara M.B., Garsky V.M., Marshall M.S., Pompliano D.L., Gibbs J.B. (1991). Sequence dependence of protein isoprenylation. J. Biol. Chem..

[12] Morris C.M., Haataja L., McDonald M., Gough S., Markie D., Groffen J., Heisterkamp N. (2000). The small GTPase RAC3 gene is located within chromosome band 17q25.3 outside and telomeric of a region commonly deleted in breast and ovarian tumours. Cytogenet. Cell Genet..

[13] Cropp C.S., Lidereau R., Campbell G., Champene M.H., Callahan R. (1990). Loss of heterozygosity on chromosomes 17 and 18 in breast carcinoma: Two additional regions identified. Proc. Natl. Acad. Sci. USA.

[14] Cornelis R.S., Devilee P., van Vliet M., Kuipers-Dijkshoorn N., Kersenmaeker A., Bardoel A., Khan P.M., Cornelisse C.J. (1993). Allele loss patterns on chromosome 17q in 109 breast carcinomas indicate at least two distinct target regions. Oncogene.

[15] Philips A., Blein M., Robert A., Chambon J.P., Baghdiguian S., Weill M., Fort P. (2003). Ascidians as a vertebrate-like model organism for physiological studies of Rho GTPase signaling. Biol. Cell.

[16] Albertinazzi C., Gilardelli D., Paris S., Longhi R., de Curtis I. (1998). Overexpression of a neural-specific Rho family GTPase, cRac1B, selectively induces enhanced neuritogenesis and neurite branching in primary neurons. J. Cell Biol..

[17] Bolis A., Corbetta S., Cioce A., de Curtis I. (2003). Differential distribution of Rac1 and Rac3 GTPases in the developing mouse brain: Implications for a role of Rac3 in Purkinje cell differentiation. Eur. J. Neurosci..

[18] Cox A.D., Der C.J. (1992). Protein prenylation: More than just glue?. Curr. Opin. Cell Biol..

[19] Joyce P.L., Cox A.D. (2003). Rac1 and Rac3 are targets for geranylgeranyltransferase I inhibitor-mediated inhibition of signaling, transformation, and membrane ruffling. Cancer Res..

[20] Corbetta S., Gualdoni S., Ciceri G., Monari M., Zuccaro E., Tybulewicz V.L., de Curtis I. (2009). Essential role of Rac1 and Rac3 GTPases in neuronal development. FASEB J..

[21] Pennucci R., Gucciardi I., de Curtis I. (2019). Rac1 and Rac3 GTPases differently influence the morphological maturation of dendritic spines in hippocampal neurons. PLoS ONE.

[22] Honkura N., Matsuzaki M., Noguchi J., Ellis-Davies G.C., Kasai H. (2008). The subspine organization of actin fibers regulates the structure and plasticity of dendritic spines. Neuron.

[23] Nestor M.W., Cai X., Stone M.R., Bloch R.J., Thompson S.M. (2011). The actin binding domain of βI-spectrin regulates the morphological and functional dynamics of dendritic spines. PLoS ONE.

[24] De Curtis I. (2008). Functions of Rac GTPases during neuronal development. Dev. Neurosci..

[25] Sugihara K., Nakatsuji N., Nakamura K., Nakao K., Hashimoto R., Otani H., Sakagami H., Kondo H., Nozawa S., Aiba A. (1998). Rac1 is required for the formation of three germ layers during gastrulation. Oncogene.

[26] Chen L., Liao G., Waclaw R.R., Burns K.A., Linquist D., Campbell K., Zheng Y., Kuan C.Y. (2007). Rac1 controls the formation of midline commissures and the competency of tangential migration in ventral telencephalic neurons. J. Neurosci..

[27] Kassai H., Terashima T., Fukaya M., Nakao K., Sakahara M., Watanabe M., Aiba A. (2008). Rac1 in cortical projection neurons is selectively required for midline crossing of commissural axonal formation. Eur. J. Neurosci..

[28] Grimsley-Myers C.M., Sipe C.W., Géléoc G.S., Lu X. (2009). The small GTPase Rac1 regulates auditory hair cell morphogenesis. J. Neurosci..

[29] Haditsch U., Leone D.P., Farinelli M., Chrostek-Grashoff A., Brakebusch C., Mansuy I.M., McConnell S.K., Palmer T.D. (2009). A central role for the small GTPase Rac1 in hippocampal plasticity and spatial learning and memory. Mol. Cell Neurosci..

[30] Haruta M., Bush R.A., Kjellstrom S., Vijayasarathy C., Zeng Y., Le Y.Z., Sieving P.A. (2009). Depleting Rac1 in mouse rod photoreceptors protects them from photo-oxidative stress without affecting their structure or function. Proc. Natl. Acad. Sci. USA.

[31] Leone D.P., Srinivasan K., Brakebusch C., McConnell S.K. (2010). The rho GTPase Rac1 is required for proliferation and survival of progenitors in the developing forebrain. Dev. Neurobiol..

[32] Tahirovic S., Hellal F., Neukirchen D., Hindges R., Garvalov B.K., Flynn K.C., Stradal T.E., Chrostek-Grashoff A., Brakebusch C., Bradke F. (2010). Rac1 regulates neuronal polarization through the WAVE complex. J. Neurosci..

[33] Dietz D.M., Sun H., Lobo M.K., Cahill M.E., Chadwick B., Gao V., Koo J.W., Mazei-Robison M.S., Dias C., Maze I. (2012). Rac1 is essential in cocaine-induced structural plasticity of nucleus accumbens neurons. Nat. Neurosci..

[34] Haditsch U., Anderson M.P., Freewoman J., Cord B., Babu H., Brakebusch C., Palmer T.D. (2013). Neuronal Rac1 is required for learning-evoked neurogenesis. J. Neurosci..

[35] Corbetta S., D’Adamo P., Gualdoni S., Braschi C., Berardi N., de Curtis I. (2008). Hyperactivity and novelty-induced hyperreactivity in mice lacking Rac3. Behav. Brain Res..

[36] Pennucci R., Tavano S., Tonoli D., Gualdoni S., de Curtis I. (2011). Rac1 and Rac3 GTPases regulate the development of hilar mossy cells by affecting the migration of their precursors to the hilus. PLoS ONE.

[37] Wonders C.P., Anderson S.A. (2006). The origin and specification of cortical interneurons. Nat. Rev. Neurosci..

[38] Nadarajah B., Alifragis P., Wong R.O., Parnavelas J.G. (2002). Ventricle directed migration in the developing cerebral cortex. Nat. Neurosci..

[39] Ang E.S., Haydar T.F., Gluncic V., Rakic P. (2003). Four-dimensional migratory coordinates of GABAergic interneurons in the developing mouse cortex. J. Neurosci..

[40] Tanaka D.H., Maekawa K., Yanagawa Y., Obata K., Murakami F. (2006). Multidirectional and multizonal tangential migration of GABAergic interneurons in the developing cerebral cortex. Development.

[41] Hernández-Miranda L.R., Parnavelas J.G., Chiara F. (2010). Molecules and mechanisms involved in the generation and migration of cortical interneurons. ASN Neuro.

[42] Vidaki M., Tivodar S., Doulgeraki K., Tybulewicz V., Kessaris N., Pachnis V., Karagogeos D. (2012). Rac1-dependent cell cycle exit of MGE precursors and GABAergic interneuron migration to the cortex. Cereb. Cortex.

[43] Vaghi V., Pennucci R., Talpo F., Corbetta S., Montinaro V., Barone C., Croci L., Spaiardi P., Consalez G.G., Biella G. (2014). Rac1 and Rac3 GTPases control synergistically the development of cortical and hippocampal GABAergic interneurons. Cereb. Cortex.

[44] Tivodar S., Kalemaki K., Kounoupa Z., Vidaki M., Theodorakis K., Denaxa M., Kessaris N., de Curtis I., Pachnis V., Karagogeos D. (2015). Rac-GTPases regulate microtubule stability and axon growth of cortical GABAergic interneurons. Cereb. Cortex.

[45] Nakamura T., Ueyama T., Ninoyu Y., Sakaguchi H., Choijookhuu N., Hishikawa Y., Kiyonari H., Kohta M., Sakahara M., de Curtis I. (2017). Novel role of Rac-Mid1 signaling in medial cerebellar development. Development.

[46] Pennucci R., Talpo F., Astro V., Montinaro V., Morè L., Cursi M., Castoldi V., Chiaretti S., Bianchi V., Marenna S. (2016). Loss of Either Rac1 or Rac3 GTPase Differentially Affects the Behavior of Mutant Mice and the Development of Functional GABAergic Networks. Cereb. Cortex.

[47] Ito H., Morishita R., Mizuno M., Tabata H., Nagata K.I. (2019). Rho family GTPases, Rac and Cdc42, control the localization of neonatal dentate granule cells during brain development. Hippocampus.

[48] Zamboni V., Jones R., Umbach A., Ammoni A., Passafaro M., Hirsch E., Merlo G.R. (2018). Rho GTPases in ntellectual Disability: From Genetics to Therapeutic Opportunities. Int. J. Mol. Sci..

[49] Reijnders M.R.F., Ansor N.M., Kousi M., Yue W.W., Tan P.L., Clarkson K., Clayton-Smith J., Corning K., Jones J.R., Lam W.W.K. (2017). Deciphering Developmental Disorders Study, Millard TH, Katsanis N, Brunner HG, Banka S. RAC1 missense mutations in developmental disorders with diverse phenotypes. Am. J. Hum. Genet..

[50] Kawazu M., Ueno T., Kontani K., Ogita Y., Ando M., Fukumura K., Yamato A., Soda M., Takeuchi K., Miki Y. (2013). Transforming mutations of RAC guanosine triphosphatases in human cancers. Proc. Natl. Acad. Sci. USA.

[51] Costain G., Callewaert B., Gabriel H., Tan T.Y., Walker S., Christodoulou J., Lazar T., Menten B., Orkin J., Sadedin S. (2019). De novo missense variants in RAC3 cause a novel neurodevelopmental syndrome. Genet. Med..

[52] Rosenberg B.J., Gil-Henn H., Mader C.C., Halo T., Yin T., Condeelis J., Machida K., Wu Y.I., Koleske A.J. (2017). Phosphorylated cortactin recruits Vav2 guanine nucleotide exchange factor to activate Rac3 and promote invadopodial function in invasive breast cancer cells. Mol. Biol. Cell.

[53] Hiraide T., Kaba Yasui H., Kato M., Nakashima M., Saitsu H. (2019). A de novo variant in RAC3 causes severe global developmental delay and a middle interhemispheric variant of holoprosencephaly. J. Hum. Genet..

[54] Etienne-Manneville S., Hall A. (2002). Rho GTPases in cell biology. Nature.

[55] Svensmark J.H., Brakebusch C. (2019). Rho GTPases in cancer: Friend or foe?. Oncogene.

[56] Gómez del Pulgar T., Benitah S.A., Valerón P.F., Espina C., Lacal J.C. (2005). Rho GTPase expression in tumourigenesis: Evidence for a significant link. Bioessays.

[57] De P., Aske J.C., Dey N. (2019). RAC1 Takes the Lead in Solid Tumors. Cells.

[58] Mira J.P., Benard V., Groffen J., Sanders L.C., Knaus U.G. (2000). Endogenous, hyperactive Rac3 controls proliferation of breast cancer cells by a p21-activated kinase-dependent pathway. Proc. Natl. Acad. Sci. USA.

[59] Hwang S., Chang J.H., Cheng T.S., Sy W.D., Lieu A.S., Lin C.L., Lee K.S., Howng S.L., Hong Y.R. (2005). Expression of Rac3 in human brain tumors. J. Clin. Neurosci..

[60] Leung K., Nagy A., Gonzalez-Gomez I., Groffen J., Heisterkamp N., Kaartinen V. (2003). Targeted expression of activated Rac3 in mammary epithelium leads to defective postlactational involution and benign mammary gland lesions. Cells Tissues Organs.

[61] Chan A.Y., Coniglio S.J., Chuang Y.Y., Michaelson D., Knaus U.G., Philips M.R., Symons M. (2005). Roles of the Rac1 and Rac3 GTPases in human tumor cell invasion. Oncogene.

[62] Keller P.J., Gable C.M., Wing M.R., Cox A.D. (2005). Rac3-mediated transformation requires multiple effector pathways. Cancer Res..

[63] Walker M.P., Zhang M., Le T.P., Wu P., Lainé M., Greene G.L. (2011). RAC3 is a pro-migratory co-activator of ERα. Oncogene.

[64] Morcillo-Garcia S., Noblejas-Lopez M.D.M., Nieto-Jimenez C., Perez-Peña J., Nuncia-Cantarero M., Győrffy B., Amir E., Pandiella A., Galan-Moya E.M., Ocana A. (2019). Genetic mutational status of genes regulating epigenetics: Role of the histone methyltransferase KMT2D in triple negative breast tumors. PLoS ONE.

[65] Gest C., Joimel U., Huang L., Pritchard L.L., Petit A., Dulong C., Buquet C., Hu C.Q., Mirshahi P., Laurent M. (2013). Rac3 induces a molecular pathway triggering breast cancer cell aggressiveness: Differences in MDA-MB-231 and MCF-7 breast cancer cell lines. BMC Cancer.

[66] Engers R., Ziegler S., Mueller M., Walter A., Willers R., Gabbert H.E. (2007). Prognostic relevance of increased Rac GTPase expression in prostate carcinomas. Endocr. Relat. Cancer.

[67] Lehr J.E., Pienta K.J. (1998). Preferential adhesion of prostate cancer cells to a human bone marrow endothelial cell line. J. Natl. Cancer Inst..

[68] Chatterjee M., Sequeira L., Jenkins-Kabaila M., Dubyk C.W., Pathak S., van Golen K.L. (2011). Individual rac GTPases mediate aspects of prostate cancer cell and bone marrow endothelial cell interactions. J. Signal. Transduct..

[69] Zhang C., Liu T., Wang G., Wang H., Che X., Gao X., Liu H. (2017). Rac3 Regulates Cell Invasion, Migration and EMT in Lung Adenocarcinoma through p38 MAPK Pathway. J. Cancer.

[70] Liu T.Q., Wang G.B., Li Z.J., Tong X.D., Liu H.X. (2015). Silencing of Rac3 inhibits proliferation and induces apoptosis of human lung cancer cells. Asian Pac. J. Cancer Prev..

[71] Lai Y.J., Tsai J.C., Tseng Y.T., Wu M.S., Liu W.S., Lam H.I., Yu J.H., Nozell S.E., Benveniste E.N. (2017). Small G protein Rac GTPases regulate the maintenance of glioblastoma stem-like cells in vitro and in vivo. Oncotarget.

[72] Hantschel O., Superti-Furga G. (2004). Regulation of the c-Abl and Bcr-Abl tyrosine kinases. Nat. Rev. Mol. Cell Biol..

[73] Hajdo-Milasinović A., Ellenbroek S.I., van Es S., van der Vaart B., Collard J.G. (2007). Rac1 and Rac3 have opposing functions in cell adhesion and differentiation of neuronal cells. J. Cell Sci..

[74] Hajdo-Milasinovic A., van der Kammen R.A., Moneva Z., Collard J.G. (2009). Rac3 inhibits adhesion and differentiation of neuronal cells by modifying GIT1 downstream signaling. J. Cell Sci..

[75] Hoefen R.J., Berk B.C. (2006). The multifunctional GIT family of proteins. J. Cell Sci..

[76] Albiges-Rizo C., Destaing O., Fourcade B., Planus E., Block M.R. (2009). Actin machinery and mechanosensitivity in invadopodia, podosomes and focal adhesions. J. Cell Sci..

[77] Artym V.V., Zhang Y., Seillier-Moiseiwitsch F., Yamada K.M., Mueller S.C. (2006). Dynamic interactions of cortactin and membrane type 1 matrix metalloproteinase at invadopodia: Defining the stages of invadopodia formation and function. Cancer Res..

[78] Abe K., Rossman K., Liu B., Ritola K.D., Chiang D., Campbell S.L., Burridge K., Der C.J. (2000). Vav2 is an activator of Cdc42, Rac1, and RhoA. J. Biol. Chem..

[79] Donnelly S.K., Cabrera R., Mao S.P.H., Christin J.R., Wu B., Guo W., Bravo-Cordero J.J., Condeelis J.S., Segall J.E., Hodgson L. (2017). Rac3 regulates breast cancer invasion and metastasis by controlling adhesion and matrix degradation. J. Cell Biol..

[80] Haataja L., Kaartinen V., Groffen J., Heisterkamp N. (2002). The small GTPase Rac3 interacts with the integrin-binding protein CIB and promotes integrin α_IIb_β_3_-mediated adhesion and spreading. J. Biol. Chem..

[81] Naik U.P., Patel P.M., Parise L.V. (1997). Identification of a novel calcium-binding protein that interacts with the integrin α_IIb_ cytoplasmic domain. J. Biol. Chem..

[82] Zhu W.L., Hossain M.S., Guo D.Y., Liu S., Tong H., Khakpoor A., Casey P.J., Wang M. (2011). A role for Rac3 GTPase in the regulation of autophagy. J. Biol. Chem..

[83] Skowyra D., Craig K.L., Tyers M., Elledge S.J., Harper J.W. (1997). F-box proteins are receptors that recruit phosphorylated substrates to the SCF ubiquitin-ligase complex. Cell.

[84] Zheng N., Schulman B.A., Song L., Miller J.J., Jeffrey P.D., Wang P., Chu C., Koepp D.M., Elledge S.J., Pagano M. (2002). Structure of the Cul1-Rbx1-Skp1-F boxSkp2 SCF ubiquitin ligase complex. Nature.

[85] Dong S., Zhao J., Wei J., Bowser R.K., Khoo A., Liu Z., Luketich J.D., Pennathur A., Ma H., Zhao Y. (2014). F-box protein complex FBXL19 regulates TGFβ1-induced E-cadherin down-regulation by mediating Rac3 ubiquitination and degradation. Mol. Cancer.

[86] Finkielstein C.V., Overduin M., Capelluto D.G. (2006). Cell migration and signaling specificity is determined by the phosphatidylserine recognition motif of Rac1. J. Biol. Chem..

